# A Treatise on General Pathology

**Published:** 1853-10

**Authors:** 


*A Treatise on General Pathology.* By Dr. T. HENLÉ, Professor of Anatomy and Physiology in Heidelberg. *Translated from the German.* By HENRY C. PRESTON, A. M., M. D. Philadelphia. Lindsay & Blakiston, 1853. pp. 391.

It is with sincere pleasure that we notice the appearance of a translation of Prof. Henlé's treatise on Pathology, for we have long felt the want of an easily accessible standard work on the subject. Prof. Henlé's reputation as an anatomist and pathologist has been long and well established—his General Anatomy, published 1841, being regarded as a classical work all over the world. The present treatise forms the first part of his extensive work on Rational Pathology, which, begun in 1846, has only recently been concluded. It is, as the author himself informs us, an attempt to collect into the same form, those physiological facts which the observations of diseased bodies have brought to light, together with the theories and hypotheses pertaining to them. This volume, or the part on General Pathology, treats of all those topics which are especially attractive to every reflecting medical man, and which for centuries have occupied the attention of the most distinguished medical minds.

Prof. H. commences his works with a description of the different medical systems. The method of cure derived from clinical experience he calls the *Empirical* system; whilst the method which considers the symptoms in their dependence upon each other, he denotes as the Physiological or *Rational* system. He deems the Empirical system preferable, provided the medical experiences be pure and undeniable: the statistical method of Louis for classing experiences, he does not regard as applicable to rare or chronic diseases, nor to those whose course presents great fluctuation.

Rational Pathology he divides into four sections:

1. The inquiry into the idea and nature of disease.

2. The doctrine of the causes of diseases in general, or General Etiology.

3. The local relations of disease and the conditions of its propagation in the organism.

4. The relation of disease in regard to time, or the general history of disease, its course, duration and termination.

These divisions are followed by an admirable historical view of medical systems grouped into seven periods.

*First* period, from 500 to 100, B. C. The period of the Pythagorean philosophy of nature. *Second* period, from 100, B. C., to the beginning of the sixteenth century after Christ, commonly under the influence of the corpuscular philosophy, and ending with the regeneration of the empirical sciences. The *third* period, from the beginning of the sixteenth to the seventeenth century, is characterised by the development of the doctrines of Theophrastus Paracelsus. The *fourth*, which occupies the first half of the seventeenth century, originates from the advancement of Chemistry and Physics. The *fifth*, extending as far as the commencement of the eighteenth century is the period of reaction against the materialism of the foregoing period by the systems of Hoffmann and Stahl, until their final overthrow by the nervous physiology, which, in the sixth period, in the second half of the eighteenth century disappeared again in its turn. The *seventh* period, the one in which we now live, Prof. Henlé regards as originated by Schelling's Philosophy of Nature, which gave rise to the so-called natural, historical, and pathologico-anatomical schools.

After a consideration of the systems, the subject proper of General Pathology is taken up. The first, second, and third chapters, are devoted to the definition of disease, the nature of disease, and to morbid processes. The next subjects considered, are the properties of Irrritation, Reaction, and Restitution. We should be glad, if our limited space permitted, to notice each chapter in succession. We can only, however, mention the most important points contained in some of them.

In the chapter on hereditary quality our author distinguishes between *generated* or *begotten* diseases, and *hereditary* or *inherited* diseases; the latter being not merely a propagation, but also a repetition of the disease of the parents. Diseases, he thinks, are inherited from both parents, but the preponderance of one side may destroy the influence of the other, and lessen and obliterate a morbid predisposition. The chapter on Sympathy and Antagonism is very interesting and important. The remarks on the structure of the nervous system are of inferior interest. We cannot refrain from noticing a very singular view expressed in the chapter on Sympathies of Cellular tissue, as to the production of cutis anserina, namely,—that it is a spasm of the skin; this must evidently have been written before the researches of Prof. Kolliker on that subject were published. Prof. Henlé admits a rhythm for all diseases, but denies anything like a crisis, which he considers a most tenacious relic of the mythical beginnings of the science of medicine. In the terminal chapter on the signs of death, he distinguishes actual death from apparent death, by the complete loss of tone and irritability, and therefore proposes irritation by electricity as the only means applicable in doubtful cases; cold he found accelerated the rigor mortis.

So much for the topics treated of in the work. The style is attractive; the language is good, but in some places rather fanciful and not very clear, as the following passage will show:

> "The devil of medicine is disease, or in more scientific language, the morbid irritant, the *materia peccans*, the penetrating noxiousness, the morbid organism. Between the medical and the theological devil, according to biblical notices and old wives' fables, there exists not a mere analogy but a complete identity; the delirious ravings of the possessed are the language of the evil spirit who possesses."

The whole manner, however, in which the subjects are treated of, and some of the mysteries of a diseased organism explained, bears evidence to its being the work of a highly talented and cultivated mind. If a love for theory may have carried the author sometimes a little beyond his subject, we must bear in mind that this fault is a national German peculiarity. The work is excellent, and will prove a great addition to our Medical Literature.

As regards the translation, we can say, after having carefully compared it with the original, that Dr. Preston has fulfilled his difficult task with zeal and care. We hope he will continue his labors, for this treatise on General Pathology forms but a part of Prof. Henlé's original work. D. C.

